# Supplementary material for: Exploring the evolution of a trade-off between vigilance and foraging in group-living organisms
Source: R Soc Open Sci. 2015 Sep 16;2(9):150135. doi: 10.1098/rsos.150135 (PMC4593673; doi:10.1098/rsos.150135)
Supplement: Supporting data and figures [file rsos150135supp1.pdf]

Supporting Information

Olson and Haley et al. (2015)

Exploring the evolution of a trade-off between vigilance and foraging in group-living organisms

SI Text

Control treatments without predation

To verify that the effects we are measuring in these experiments are indeed caused by predation, we ran a set of control treatments without predation. Shown in Figure S1, no treatment populations evolve vigilance nor gregarious foraging behavior when there are no predators, and instead evolve to forage 100% of the time (indicated by the 5,000 fitness scores). These findings confirm that predation is indeed the primary selection pressure on the evolving prey populations.

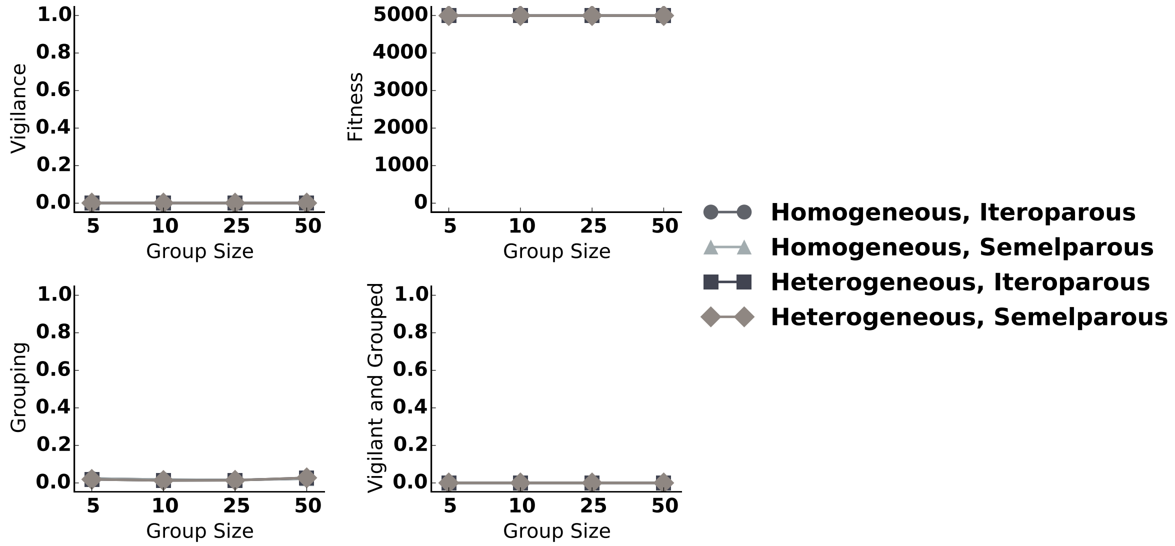

Figure S1: Foraging, grouping, and vigilance behavior in prey not experiencing predation. Error bars indicate bootstrapped 95% confidence intervals over 100 replicates; some error bars are too small to be visible.

Predator attack rate

Attack rate is measured in terms of the number of attacks per individual per simulation. Thus, an attack rate of 1 for a group size of 10 means that 10 attacks will occur on average in a 5,000 update simulation, meaning an attack will occur roughly every 500 updates. An attack rate of 5 for the same group size would occur every 100 updates, meaning that increasing the attack rate dramatically increases the number of attacks that the prey experience.

We measured the effect of the predator's attack rate to explore whether our findings are dependent on a particular attack rate. Shown in Figure S2, when prey that are forced to group in the homogeneous/semelparous treatment experience higher rates of predation (Rate 5), they evolve higher levels of vigilance because the predator can consume a large portion of the group during their lifetime. In contrast, at lower rates of predation (Rate 1), the predator attacks so infrequently that the prey instead focus more on foraging. These findings verify that predator attack rate plays a significant role in the evolution of gregarious foraging behavior via the many eyes effect.

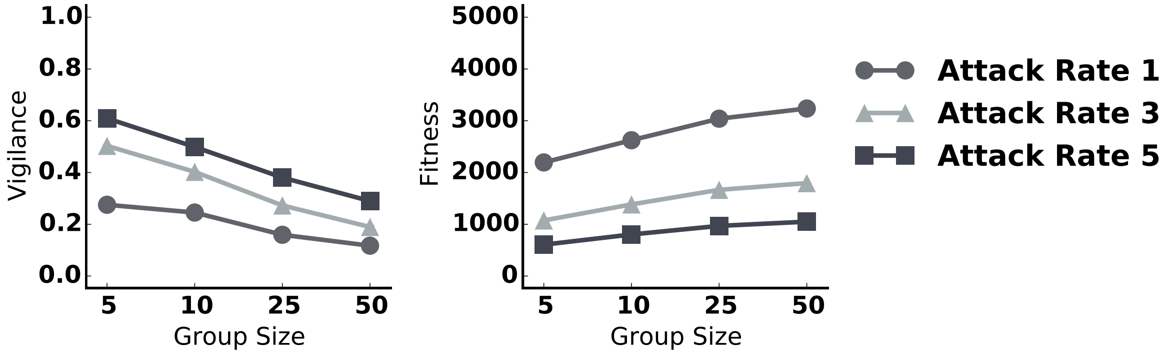

Figure S2: Foraging and vigilance behavior of prey in the homogeneous/semelparous treatment experiencing differing levels of predation. Error bars indicate bootstrapped 95% confidence intervals over 100 replicates; some error bars are too small to be visible.
